# A period of immobility after remifentanil administration protects from nausea: an experimental randomized cross-over study

**DOI:** 10.1186/s12871-016-0263-5

**Published:** 2016-10-10

**Authors:** Fabian Heuser, Christian M. Schulz, Alexander Hapfelmeier, Nadine Lehnen, Eberhard F. Kochs, Klaus J. Wagner

**Affiliations:** 1 Department of Anesthesiology and Intensive Care, Asklepios Stadtklinik Bad Tölz, Bad Tölz, Germany; 2 Department of Anesthesiology, Klinikum rechts der Isar, Technische Universität München, Munich, Germany; 3 Institute of Medical Statistics and Epidemiology, Klinikum rechts der Isar, Technische Universität München, Munich, Germany; 4 Department of Neurology, Munich University Hospital, Munich, Germany

**Keywords:** Remifentanil, Opioid, Nausea, Vomiting, PONV, Vestibulo-ocular reflex, Motion sickness

## Abstract

**Background:**

The opioid remifentanil induces a decrease of vestibulo-ocular reflex function, which has been associated with nausea and vomiting when the subjects are moved. The study investigates in healthy female volunteers if immobility after remifentanil administration protects from nausea and vomiting.

**Methods:**

In volunteers, a standardized movement intervention (a manually applied head-trunk movement forward, backward and sideward) was started 5 min (session A), 35 min (session B) or 60 min (session C) after cessation of a remifentanil infusion (0.15 μg · kg^−1^ · min^−1^). In a cross-over design, 16 participants were randomized to the early (sessions A and B) or the late intervention group (sessions A and C). Nausea was assessed using a 11-point numerical rating scale before and after each movement intervention. Differences within and between groups were assessed with non-parametric tests for paired and unpaired data.

**Results:**

Comparing sessions A, B and C, intensity of nausea was time-dependent after cessation of remifentanil administration (*p* = 0.015). In the early intervention group, nausea decreased from median 5.0 [IQR 1.5;6.0] in session A to 2.0 [1.0;3.0] in session B (*p* = 0.094); in the late intervention group nausea decreased from 3.5 [2.0;5.0] in session A to 0.5 [0.0;2.0] in session C (*p* = 0.031).

**Conclusions:**

In summary, in young healthy women, immobility after remifentanil administration protects from nausea and vomiting in a time-dependent manner. In analogy to motion sickness, opioid-induced nausea and vomiting in female volunteers can be triggered by movement.

**Trial registration:**

German Clinical Trials Register DRKS00010667. The trial was registered retrospectively on June, 20th 2016.

## Background

Opioids are a very important component of the treatment of various pain conditions including moderate and severe pain, malignant and non-malignant pain as well as chronic and acute pain [1, 2]. Opioids are the third among the top therapeutic classes by prescriptions [3] and are used during general anaesthesia, which is performed several hundred million times a year in the world [4]. The major reason for reduced patient compliance and discontinuation of opioid analgesic treatment are gastrointestinal side effects (i.e., nausea, vomiting and constipation) along with side affects regarding the central nervous system [5]. The range of incidence of nausea and vomiting in patients treated with opioids for chronic pain is reported between 10 and 50 % [6–9]. Accordingly, opioids have been identified as an independent risk factor for the development of postoperative nausea and vomiting (PONV) [10]. The incidence of PONV is approximately 20–30 % in the general population [11] which increases up to 80 % in high-risk patients [10].

Different pathways of the emetogenic effects of opioids are still under debate. Predominantly, a direct influence of opioids in the chemoreceptor trigger zone (CTZ) in the area postrema by stimulating specific opioid receptors has been discussed [11]. From there, efferent fibers reach the vomiting center, along with input from three other major areas, namely: the gastrointestinal tract, the cerebral cortex and the vestibular apparatus [12]. The vestibular system has been identified to contribute to PONV and this is also reflected by the simplified risk score for predicting PONV after general anaesthesia [10]. Recently, we showed that the opioid remifentanil induces a decrease of vestibulo-ocular reflex (VOR) function [13]. The calculated half-life time of the recovery of the VOR function after cessation of the remifentanil administration was 5.3 min [13]. During opioid administration, movement has been found to trigger nausea, while immobility protected from nausea. Therefore we suggested that the intersensory mismatch caused by the impaired VOR promotes nausea and vomiting.

To provide further evidence that PONV is, at least in part, based on this pathophysiologic principle, we hypothesized that the incidence and severity of nausea depends on the time interval between the stop of remifentanil administration and a predefined movement intervention.

## Methods

### Study design

After approval by the Ethics Committee (2411/09, September 26th, 2011) of the Faculty of Medicine of the Technische Universität München, the study was conducted with sixteen healthy young women in a randomized cross-over design. This design was chosen to rule out familiarization effects in the participants (mean age 25.8 ± 2.3 years). With the aim of building a sample at high risk for PONV, only women were invited to participate. According to the declaration of Helsinki [14], all subjects provided written informed consent and were free to withdraw from the experiment at any time of the investigation.

During each trial, subjects were laying on a stretcher in a supine position with the upper part of the body elevated at 45°. A patient monitor was used for the surveillance of the vital signs including electrocardiogram, non-invasive assessment of blood pressure in 5-min intervals and pulse oximetry. The head was stabilized with a tape in order to avoid any movement of the head. After placement of an intravenous line in an antecubital vein, continuous administration of the opioid remifentanil was started using a syringe pump (B. Braun, Melsungen, Deutschland) with a rate of 0.15 μg · kg^−1^ · min^−1^. Remifentanil was chosen due to its well-known pharmacokinetic characteristics and a steady-state plasma level after a short time of continuous intravenous administration (90 % after 17 min). The context-sensitive half-life time is 3.7 min after stopping the drip [15]. After 30 min, the administration of remifentanil was stopped.

**Fig. 1:**
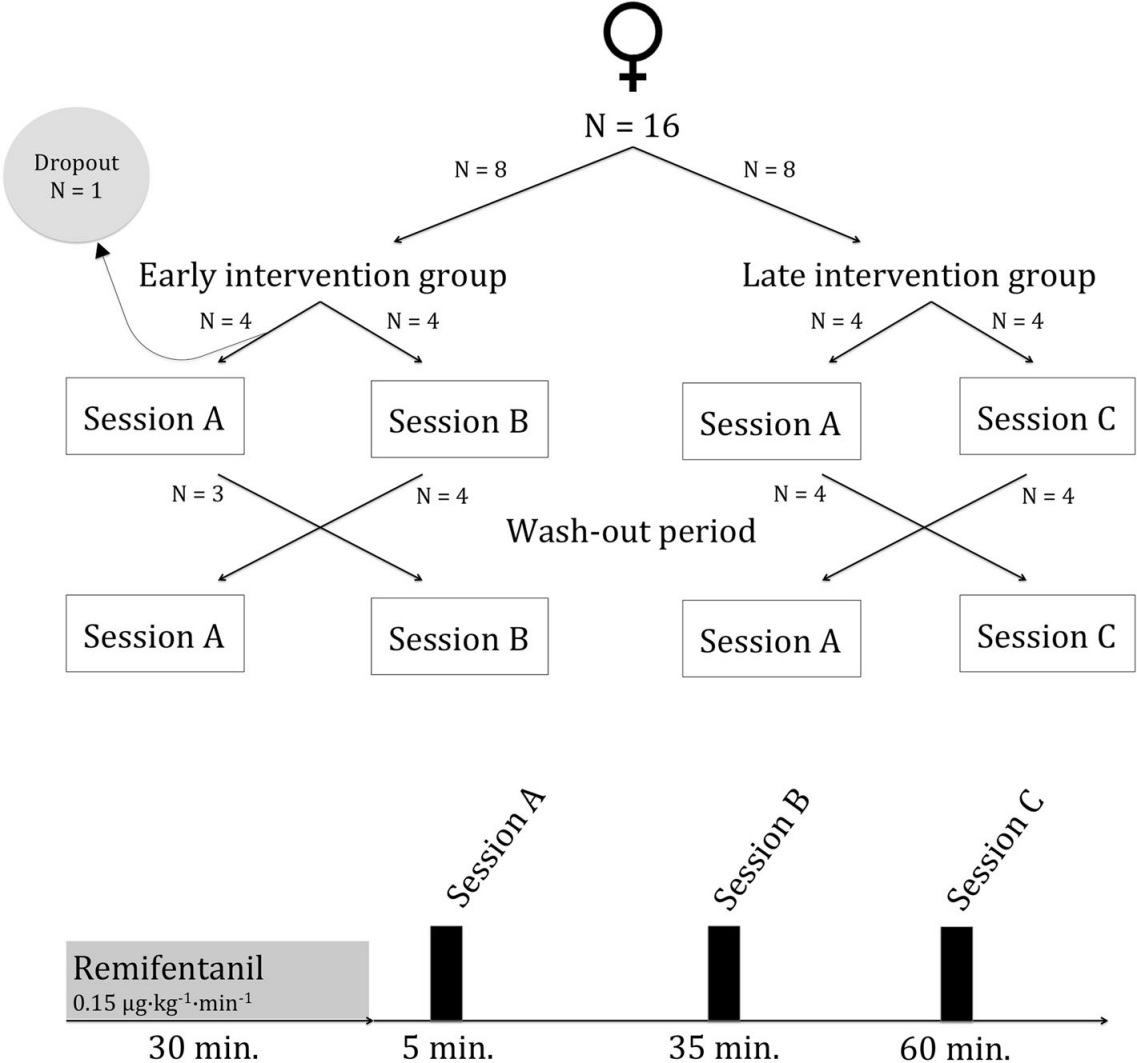
Study design

The participants were randomized into two groups (early intervention vs. late intervention). Three different sessions (A, B, C), combined in a cross-over design, were conducted. There was at least a one-day wash-out period between the measurements (Fig. 1).

In session A, the tape stabilizing the head was removed 5 min after stopping the administration of remifentanil and every subject’s head-trunk was bent manually in ± 45° forward, backward and sideward twenty times with a frequency of 1 Hz. In session B, the participants were moved identically but the intervention was started 35 min after stopping the administration of remifentanil. In session C, the same movement intervention took place 60 min after stopping the administration of remifentanil. The time periods of 35 min and 60 min were chosen because of the pharmacokinetic properties of remifentanil. The plasma concentrations are decreasing over the time and the concentration at 60 min is not be predicted to have clinically relevant effects [15]. The sessions differed only in the time interval (5 vs. 35 and 5 vs. 60 min) between stopping the administration of remifentanil and the movement intervention. In all sessions, subjects laid in a semirecumbent position, the head-stabilizing tape was removed immediately prior to the movement intervention.

### Assessment of nausea

Maximum nausea scores (experienced at the relevant time period) were noted in arbitrary values on a numerical rating scale (NRS) ranging from 0 (“everything okay”) to 10 (“vomiting”). The 11-point scale was chosen in analogy to Apfel et al. [16] and our previous work [13]. The participants rated their maximum nausea just before remifentanil initiation (NRS_0_), for the period of 30 min during opioid administration (NRS_Remi_) until just before movement, and for the period of 60 min after one of the movement interventions (NRS_post_ for sessions A, B, C).

### Subjects

The subjects did not suffer from balance disorders, headache or any other neurological diseases. They were not taking any medication, explicitly no opioids. Because of its ubiquitous use in anesthesiology to predict the risk for PONV [10], we used the Apfel-Score and included only volunteers which were classified to be part of a high risk population. Subjects were requested to fast for 6 h and to abstain from alcohol and smoking for more than 24 h before the tests. They received financial compensation for participating in the study.

### Statistical analysis

To further provide evidence that moving the subjects triggers nausea we compared nausea before the movement trigger (NRS_0_, NRS_Remi_) with nausea for those trials where the subjects were moved immediately after stopping remifentanil administration (NRS_post_). To test the hypothesis that the immobility after remifentanil administration protects from developing nausea and vomiting, we compared nausea of the trials with immediate movement (NRS_post,_ session A) with those trials where subjects were moved after 35 and 60 min after stopping remifentanil administration (session B, C). The distribution of continuous data is presented by median and interquartile range (IQR). Corresponding hypothesis testing across study groups was performed by Mann-Whitney-U and Kruskal-Wallis tests. Paired samples within study groups were compared by means of Wilcoxon signed rank tests. All statistical testing was performed on two-sided exploratory 5 % significance levels. Computations were conducted with SPSS (SPSS Statistics for Windows, Version 22.0. Armonk, NY) and R system for statistical computing [17].

## Results

The 16 participants were randomized to the groups (Fig. 1). One subject terminated the participation before session A was completed. The two groups were comparable referring to age. According to the inclusion criteria, each subject had a PONV risk of 61 % [10]. No subject indicated nausea before any trial (*n* = 30, NRS_0_ = 0 throughout). During 30 min of remifentanil administration (NRS_Remi_), a small increase was detected (*n* = 30, Median [IQR] NRS_Remi_ = 0.5 [0.0;1.3]).

### Nausea after movement intervention

**Fig. 2:**
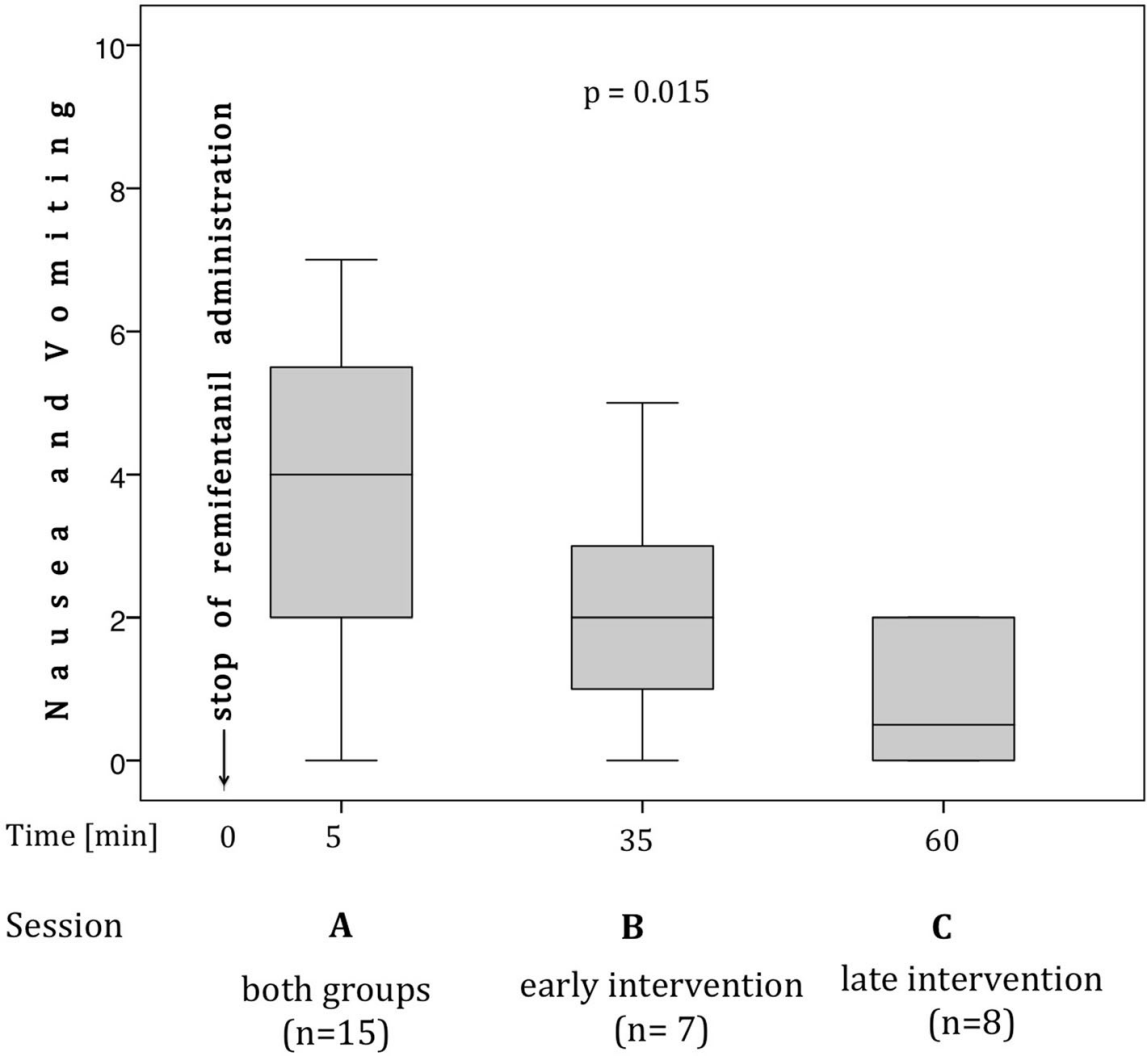
Differences in participants’ perceived nausea between the sessions A, B and C. Box plots represent medians, quartiles and range. Nausea and vomiting given as values on a numerical rating scale ranging form 0 to 10

When subjects were moved 5 min after stopping remifentanil administration (Session A, *n* = 15), they indicated significantly more nausea (Median [IQR] NRS_post_ = 4.0 [2.0;6.0]) than before movement (Median [IQR] NRS_Remi_ = 0.0 [0.0;2.0], *p* < 0.001). When comparing Session A, B and C, which are characterized by an increasing time interval between the stop of remifentanil administration and the movement intervention, the subjects indicated less nausea (*p* = 0.015, Fig. 2).

**Fig. 3:**
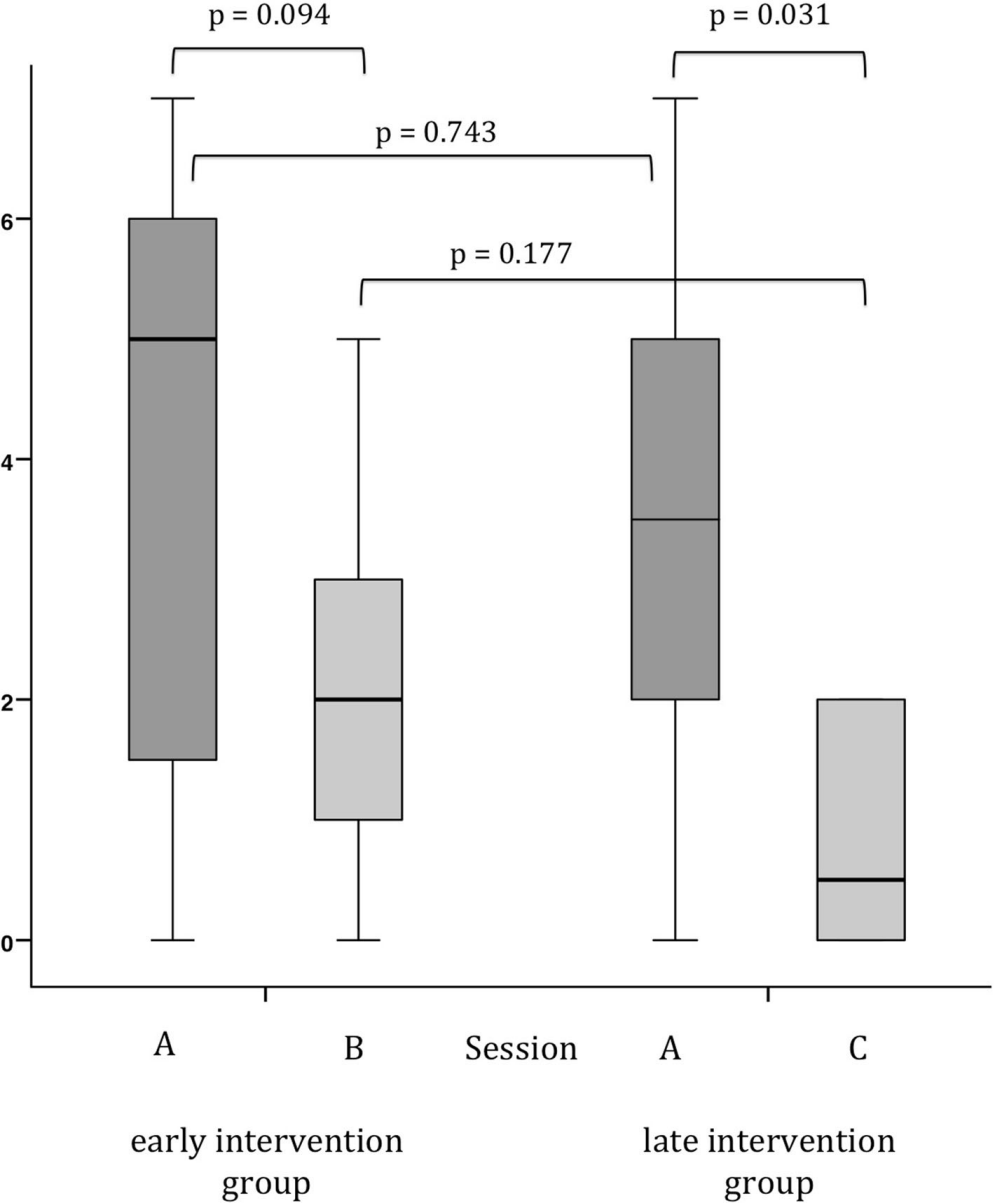
Differences in participants’ perceived nausea between the sessions A, B and C. Box plots represent medians, quartiles and range. Nausea and vomiting given as values on a numerical rating scale ranging form 0 to 10

When subjects were moved 35 or 60 min (Session B or C, respectively) after stopping the remifentanil drip, they indicated less nausea compared to their own control at the immediate intervention in session A. In the early intervention group, nausea decreased from 5.0 [1.5;6.0] in session A to 2.0 [1.0;3.0] in session B; in the late intervention group the nausea decreased from 3.5 [2.0;5.0] in session A to 0.5 [0.0;2.0] in session C (Fig. 3).

## Discussion

The present study indicates that there may be a time interval after the administration of the opioid remifentanil, during which head immobility protects from nausea. The more time the participants’ head rested immobile between administration of remifentanil and the movement intervention the less nausea was indicated (Fig. 2). This is probably due to the recovery of the VOR gain from remifentanil-induced depression, which has been shown to reflect the pharmacokinetics of the drug [13]. In our previous work we suggested that this decrease in VOR evokes a perceptual mismatch of multisensory input when the head is moved, which results in nausea [13]. This pathophysiologic principle is further supported by the present study. As in our previous work, moving patients triggered nausea during remifentanil administration.

Consistently, we found a continuous decrease of nausea between the session A and B or C, respectively, in the intra-group analysis. Some results did not provide statistical significance, specifically, the difference in the early intervention group between the sessions A and B (*p* = 0.094), and the inter-group comparison between the sessions B and C (*p* = 0.067). As the trends were in line to what was expected according to the investigated pathophysiological principle, we suggest that this is due to the small sample size.

As in our prior study, even before any movement, participants indicated slightly more nausea (Median NRS_Remi_ = 0.5) than before initiating the administration of remifentanil (NRS_0_ = 0). We suggest that this increase is of minor clinical importance and might be attributable to combined effects on the CTZ and the vestibular system [11, 13]. Generally, the nausea indicated was relatively high compared to what the PONV score predicted. This is not surprising in light of the movement intervention, which possibly overestimates the motion-induced mechanism, because the movement stimuli were of high intensity.

Our findings raise further questions with respect to the underlying pathophysiologic principles. First, do other μ-agonists similarly impact the VOR or do different opioids modulate the vestibular system differently, which could explain less nausea after opioid rotation in some patients? [18–20] Currently, there are different explanations for an improved tolerance of an opioid therapy after rotation, e.g. reduction of the opioid dosing [18], or changes in the receptor-effector relationships during a prolonged morphine exposure [21]. Second, it is of interest whether patients suffering from chronic pain treated with opioids adapt to a compromised VOR or whether the VOR itself recovers over time even under opioid treatment. In chronic pain patients, the number of patients indicating nausea after beginning a treatment with opioids, ranges from 10 to 50 % [6–9]. After a few days, an antiemetic therapy can be withdrawn as a consequence of tolerance to this side effect [22]. These patients are able to adapt (as opposed to constipation which normally requires on-going treatment). This may be similar to motion sickness where habituation is considered to be an important mechanism of adaption in case of continuous intersensory mismatch [23, 24]. This habituation is slowed by scopolamine, which has been suggested to inhibit the normal activation of the central nervous system and thus, to prompt compensatory reactions [24]. This leads to the third question that is whether some antiemetics including scopolamine, a drug for prevention of motion sickness, has its beneficial effects for the treatment of PONV through its effects on the vestibular system [25, 26].

Given the analogies between the pathophysiologic principle investigated here and the mismatch theory of motion sickness, it is not surprising that there is an increased incidence of PONV after certain types of surgery including strabismus surgery, which itself can cause a intersensory mismatch [10, 27]. A history of motion sickness itself has been identified as an independent predictor of PONV as represented in Apfel’s risk score for PONV [10]. Therefore, the findings of our study might be of clinical relevance in settings where general anaesthesia including remifentanil is administered and where post-operative pain is quite low and controlled well with non-opioid analgesics. Furthermore, the results of the present study may explain why patients in the increasing sector of outpatient surgery with immediate or very early mobilization suffer from PONV more frequently than patients in inpatient settings [28].

This study was not designed to identify a specific time interval during which immobility protects patients from PONV. It remains to investigate this hypothesized time interval in the future. Furthermore, such a time interval of vulnerability to PONV should be adapted to the individual susceptibility for PONV. Consequently, it would justify a recommendation not to move the patients after specific procedures.

Some limitations have to be considered. The sample size in this exploratory approach was relatively small and only women were tested in order to investigate a sample with high risk for PONV. Future studies will reveal whether also men would benefit from a immobility period after administration of remifentanil.

## Conclusions

This study in volunteers receiving only remifentanil showed that a time interval of immobility after remifentanil administration might protect from nausea and vomiting in a population at high risk for PONV. The results provide further evidence that the origin of PONV is multifactorial including opioid effects on the CTZ and the vestibular system. For the recommendation of a specific time interval for e.g. outpatient settings, larger studies in clinical settings are required.

## Abbreviations

CTZ: Chemoreceptor trigger zone
IQR: Interquartile range
NRS: Numerical rating scale
PONV: Postoperative nausea and vomiting
VOR: Vestibulo-ocular reflex
